# Fucoidan from *Macrocystis pyrifera* Has Powerful Immune-Modulatory Effects Compared to Three Other Fucoidans

**DOI:** 10.3390/md13031084

**Published:** 2015-02-19

**Authors:** Wei Zhang, Tatsuya Oda, Qing Yu, Jun-O Jin

**Affiliations:** 1 Shanghai Public Health Clinical Center, Shanghai Medical College, Fudan University, Shanghai 201508, China; E-Mail: weiwei061215@126.com; 2 Division of Biochemistry, Faculty of Fisheries, Nagasaki University, 1-14 Bunkyo-machi, Nagasaki, Nagasaki 852-8521, Japan; E-Mail: t-oda@nagasaki-u.ac.jp; 3 Department of Immunology and Infectious Diseases, The Forsyth Institute, 245 First Street, Cambridge, MA 02142, USA; E-Mail: qyu@forsyth.org

**Keywords:** fucoidans, neutrophils, NK cells, dendritic cells, T cells, adjuvant

## Abstract

Fucoidan, a sulfated polysaccharide purified from brown algae, has a variety of immune-modulation effects, such as promoting activation of dendritic cells (DCs), natural killer (NK) cells and T cells, and enhancing anti-viral and anti-tumor responses. However, the immune-modulatory effect of fucoidan from different seaweed extracts has not been thoroughly analyzed and compared. We analyzed fucoidans obtained from *Ascophyllum nodosum* (*A. nodosum*), *Macrocystis pyrifera* (*M. pyrifera*), *Undaria pinnatifida* (*U. pinnatifida*) and *Fucus vesiculosus* (*F. vesiculosus*) for their effect on the apoptosis of human neutrophils, activation of mouse NK cells, maturation of spleen DCs, proliferation and activation of T cells, and the adjuvant effect *in vivo*. Fucoidans from *M. pyrifera* and *U. pinnatifida* strongly delayed human neutrophil apoptosis at low concentration, whereas fucoidans from *A. nodosum* and *F. vesiculosus* delayed human neutrophil apoptosis at higher concentration. Moreover, fucoidan from *M. pyrifera* promoted NK cell activation and cytotoxic activity against YAC-1 cells. In addition, *M. pyrifera* fucoidan induced the strongest activation of spleen DCs and T cells and ovalbumin (OVA) specific immune responses compared to other fucoidans. These data suggest that fucoidan from *M. pyrifera* can be potentially useful as a therapeutic agent for infectious diseases, cancer and an effective adjuvant for vaccine.

## 1. Introduction

Fucoidans are polysaccharides containing substantial contents of l-fucose and sulfate ester groups, which are constituents of brown seaweed [1]. Fucoidans from several species of brown seaweed, such as *Fucus vesiculosus*, have a complex chemical composition, which contains decides fucose and sulfate, other monosaccharides (mannose, galactose, glucose, xylose, *etc.*) uronic acids, and even acetyl groups and protein [2]. Fucoidans are a heterogeneous assemblage with regard to their molecular mass, mono-saccharide composition, and number of sulfate and acetyl groups [2]. Fucoidans show a wide spectrum of biological effects, such as anti-coagulant and anti-thrombotic properties, anti-viral, anti-tumor, immunomodulatory, anti-oxidant, and anti-complement functions [2,3].

Several immunomodulatory effects of fucoidans have been investigated in different experimental models [2,3,4]. Fucoidan from *Fucus vesiculosus* (*F. vesiculosus*) has been well-investigated for its immunomodulatory effect, such as that on DC maturation and T cell activation [4,5,6]. Fucoidan from *F. vesiculosus* has been shown to induce macrophage activation [7]. Moreover, we have previously shown that fucoidan form *F. vesiculosus* functions can function as an effective adjuvant [4]. In addition, fucoidan from *Undaria pinnatifida* (*U. pinnatifida*) promotes T cell and natural killer (NK) cell activation, resulting in enhancement of pro-inflammatory cytokine production [8]. Fucoidan from *Ascophyllum nodosum* (*A. nodosum*) induces high level of nitric oxide (NO) production from mouse macrophage cell line RAW264.7 cells [9]. Based on these observations and other previous findings, it has been proposed that fucoidans may represent promising candidates as effective immune activators. However, differences in the immunomodulatory effect of fucoidans from different species have not been compared.

In this study, we compared the immune modulatory effect of four fucoidans from *A. nodosum*, *Macrocystis pyrifera* (*M. pyrifera*), *U. pinnatifida* and *F. vesiculosus* including their effect on human neutrophil apoptosis and on mouse NK cell, DC and T cell activation *in vivo*, as well as their adjuvant effect on antigen-specific immune responses. This study aims to identify the most immune active fucoidan to be studied further as potential novel drugs for infectious diseases, cancer and effective adjuvant for vaccine.

## 2. Results and Discussion

### 2.1. Chemical Composition of the Fucoidans

Fucoidan basically contains fucose (Fuc) as a main component and xylose (Xyl), glucose (Glu), Mannose (Man), and galactose (Gal) as minor composions [2]. The chemical composition of four fucoidans under investigation is summarized in Table 1. The monosaccharide composition profile of four fucoidans fraction was similar, whereas fucoidan from *U. pinnatifida* had higher galactose content than other fucoidans. Moreover, the four fucoidans had similar sulfate half-ester levels, whereas the levels of uronic acid in fucoidan from *M. pyrifera* and *F. vesiculosus* were much higher than the other two fucoidans (Table 1).

**Table 1:** Compositions (%) of fucoidans from various sources (*w*/*w*).

Fucoidan Source	Composition of Neutral Sugar ^a^					UA ^b^	SO_4_^2−^ ^c^
	Fuc	Xyl	Glu	Man	Gal		
*Ascophyllum nodosum*	39.80	3.68	0.88	0.72	3.37	1.72	24.07
*Macrocystis pyrifera*	25.77	0.84	1.14	1.12	3.93	5.54	27.32
*Undaria pinnatifida*	28.27	0.45	0.49	0.30	24.94	1.89	29.90
*Fucus vesiculosus*	38.02	2.73	0.49	1.27	3.38	5.49	24.53

^a^ Determined by HPLC assay after acid hydrolysis; ^b^ Determined by carbazole method and calculated as glucuronic acid equivalent; ^c^ Determined by turbidimetric assay after acid hydrolysis.

### 2.2. Effect of Fucoidans on Apoptosis and Pro-Inflammatory Cytokine Production in Human Neutrophils

**Figure 1:**
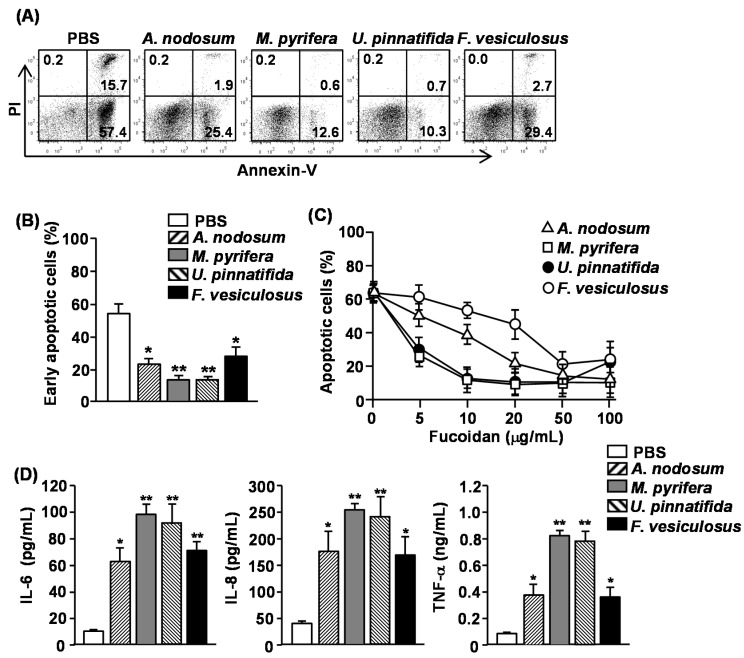
Effect of fucoidans on spontaneous apoptosis and pro-inflammatory cytokine production of human neutrophils. Isolated peripheral blood neutrophils (2 × 10^5^) were cultured with or without fucoidans (10 μg/mL) for 24 h. (**A**) Cell apoptosis was assessed by Annexin V-FITC and PI staining. Numbers in the plots show the percentage of the cells in the respective quadrant among the total cells shown in the plots; (**B**) Percentage of early apoptotic cells (Annexin V^+^PI^−^ cells) was shown. Data are representative or the average of analyses of five samples from five donors for each group; (**C**) Dose-dependent delay of neutrophil apoptosis by fucoidans was measured by morphological changes. Data are the average of analyses of five samples from five donors; (**D**) IL-6, IL-8 and TNF-α concentrations in the cultured supernatants were measured by ELISA. Data are the average of analyses of 5 samples from 5 donors. Data shown are the mean ± SEM. * *p <* 0.05; ** *p <* 0.01 *versus* PBS (phosphate buffered saline) group.

Numerous studies have shown that neutrophil apoptosis can be delayed or accelerated by cytokines and other inflammatory mediators [10,11,12]. Moreover, our recent study showed that fucoidans from *U. pinnatifida* delayed neutrophil apoptosis [13]. We therefore assessed whether other fucoidans also have similar effect on human neutrophils. Since a common marker of apoptotic cells is phosphatidyl serine (PS) exposure on the outer leaflet of the plasma membrane, we measured PS exposure by Annexin V staining and flow cytometry to identify the apoptotic cells. In addition, cells were simultaneously stained with propidium Iodide (PI) to identify the late-apoptotic or necrotic cells with membrane that is permeable to PI. Purified human neutrophils were incubated in the presence or absence of 50 μg/mL fucoidan and apoptosis was measured. More than 70% of neutrophils showed spontaneous apoptosis as indicated by positive Annexin V staining after 24 h of culture (Figure 1A). All fucoidans markedly reduced the percentage of Annexin V^+^PI^−^ cells, which were cells in the early stage of apoptosis, indicating that they inhibited apoptotic cell death (Figure 1A,B). The most potent inhibitors were fucoidan from *M. pyrifera* and *U. pinnatifida*, which reduced neutrophil apoptosis by more than 80% as compared to controls, whereas fucoidans from *A. nodosum* and *F. vesiculosus* reduced neutrophil apoptosis by approximately 50%. We next assessed the dose-dependent effect of four different fucoidans on neutrophil apoptosis. Fucoidan from *M. pyrifera* or *U. pinnatifida* showed a considerable, dose-dependent inhibiting effect on neutrophil apoptosis at concentrations between 5–100 μg/mL, whereas those from *A. nodosum* and *F. vesiculosus* only prevented neutrophil apoptosis at concentration of 50–100 μg/mL (Figure 1C).

Activated neutrophils can survive much longer than non-activated cells and can produce a number of inflammatory chemokines and cytokines [10,14,15]. We therefore assessed whether fucoidan stimulation in human neutrophil can induce the production of pro-inflammatory cytokines from human neutrophils. Purified human neutrophils were treated with 50 μg/mL fucoidans. After 24 h of culture, the concentrations of interleukin (IL)-6, IL-8 and tumor necrosis factor-α (TNF-α) in the culture medium were measured. All fucoidans significantly increased the production of IL-6, IL-8 and TNF-α from neutrophils. Consistent with the delay of apoptosis, fucoidans from *M. pyrifera* or *U. pinnatifida* induced the highest amount of cytokine production. These data demonstrated that fucoidans delay the apoptosis and promote pro-inflammatory cytokine production in human neutrophils, and fucoidans from *M. pyrifera* and *U. pinnatifida* have the strongest effect on both apoptosis and cytokine production.

### 2.3. Effect of Fucoidans on the Activation and Cytotoxicity of NK Cells

**Figure 2:**
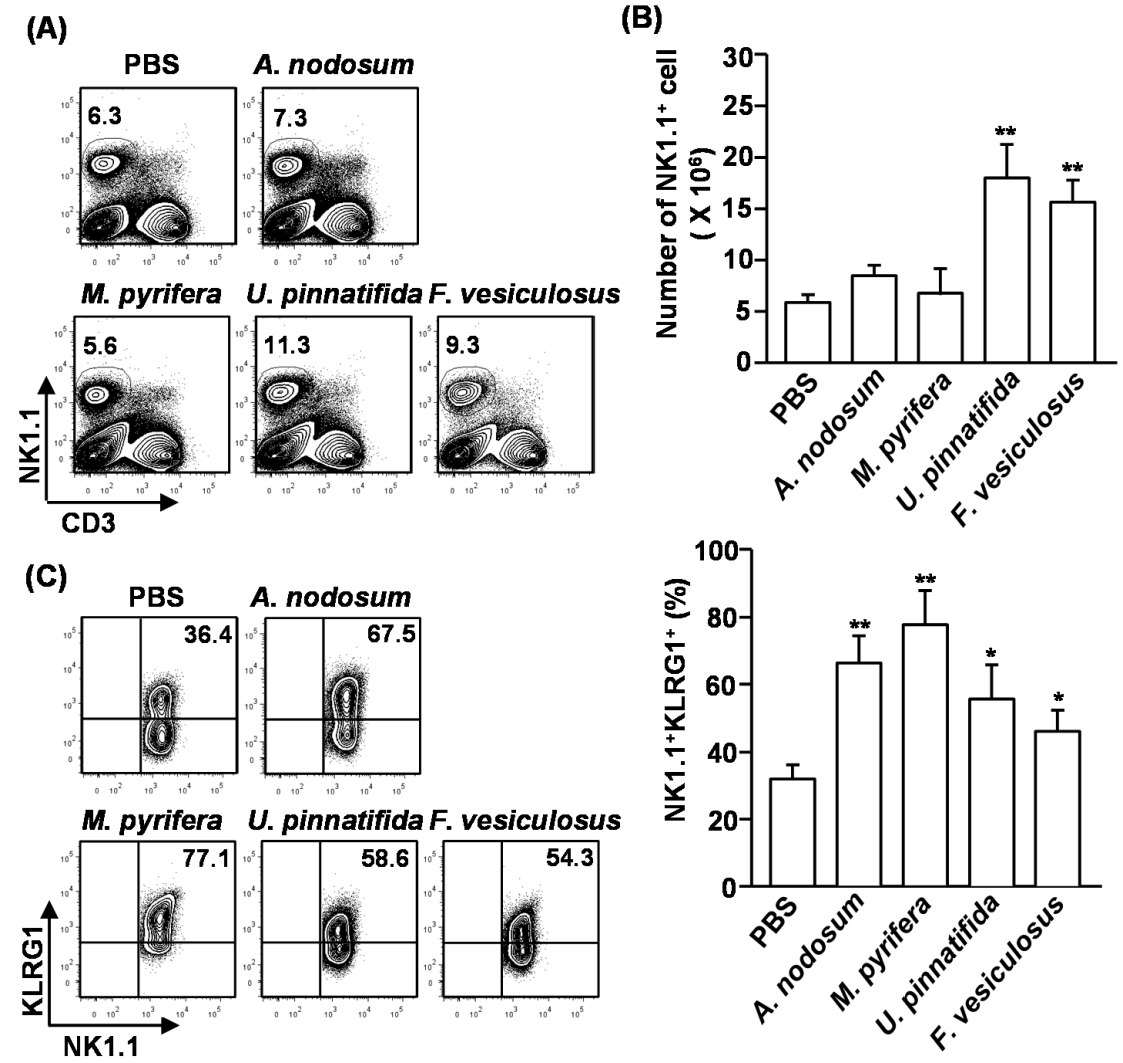

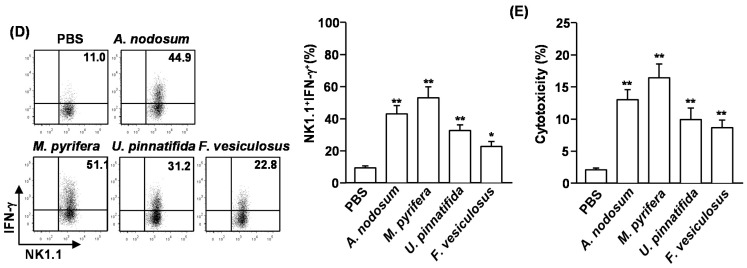
Fucoidans promote activation and maturation of mouse NK cells *in vivo*. C57BL/6 mice were injected with fucoidans (50 mg/kg) for four consecutive days. (**A**) Frequency of NK cells, defined as CD3^−^NK1.1^+^, in the spleen is indicated by the number shown in each plot; (**B**) Absolute number of NK cells in the spleen; (**C**) Expression levels of KLRG1 in CD3^−^NK1.1^+^ cells (left panel). Mean percentage of NK1.1^+^KLRG1^+^ cells (right panel); (**D**) Percentage of IFN-γ-producing cells among CD3^−^NK1.1^+^ cells in the spleen was assessed by flow cytometric analysis cells (left panel). Mean percentage of NK1.1^+^IFN-γ^+^ cells (right panel); (**E**) CD3^−^NK1.1^+^ cells were cultured with YAC-1 target cells at an E:T ration of 10:1 for 4 h. Percent specific lysis was measured as described at Method. All data are representative of or the average of analyses of six independent samples (2 mice per experiment, total 3 independent experiments). Data shown are the mean ± SEM. * *p <* 0.05; ** *p <* 0.01 *versus* PBS group.

NK cells play crucial roles in cell-mediated immunity and elimination of tumor cells. Since polysaccharide can induce activation and cytotoxicity on NK cells, we assessed whether fucoidans also can promote NK cell activation and cytotoxicity. Moreover, previous study showed 50 mg/kg of fucoidan from *F. vesiculosus* induced NK cell activation mouse *in vivo* [16], we injected *intraperitoneally* (*i.p.*) C57BL/6 mice with 50 mg/kg of fucoidans for four successive days and the percentages and cell numbers of CD3^−^NK1.1^+^ cells in the spleen were measured. Upon administration of fucoidans from *U. pinnatifida* and *F. vesiculosus*, the percentages and cell numbers of CD3^−^NK1.1^+^ cells were significantly increased compared to the controls. In contrast, the other two fucoidans had no effect (Figure 2A,B). Next, we examined the activation of NK cells after fucoidan injection. Killer cell lectin-like receptor subfamily G member 1 (KLRG1), which is the terminal differentiation and maturation marker in NK cells, was substantially up-regulated in NK cells that were treated with all type of fucoidans for 4 days (Figure 2C). Moreover, intracellular interferon-γ (IFN-γ) levels, which is normally produced by activated NK cells, were markedly increased in NK cells by fucoidan treatment (Figure 2D). Interestingly, fucoidan from *M. pyrifera* showed the strongest effect on the maturation and activation of NK cells, although it did not increase the number of NK cells in spleen.

We then assessed whether systemic administration of fucoidans can induce cytotoxic activity in NK cells against YAC-1 cells, which are sensitive to lysis by activated murine NK cells. CD3^−^NK1.1^+^ cells were isolated from spleen of fucoidan-treated mice and co-cultured with YAC-1 cells for 4 h. All fucoidans induced significant increases in the cytotoxic activity of NK cells against YAC-1 cells. Consistent with the activation and maturation of NK cells, fucoidan from *M. pyrifera* induced the highest cytotoxicity in NK cells against YAC-1 cells (Figure 2E). These results indicate that systemic administration of fucoidans promotes NK cell activation and cytotoxicity.

### 2.4. Effect of Fucoidans on the Activation and Maturation of Spleen Dendritic Cells (DCs) in Vivo

**Figure 3:**
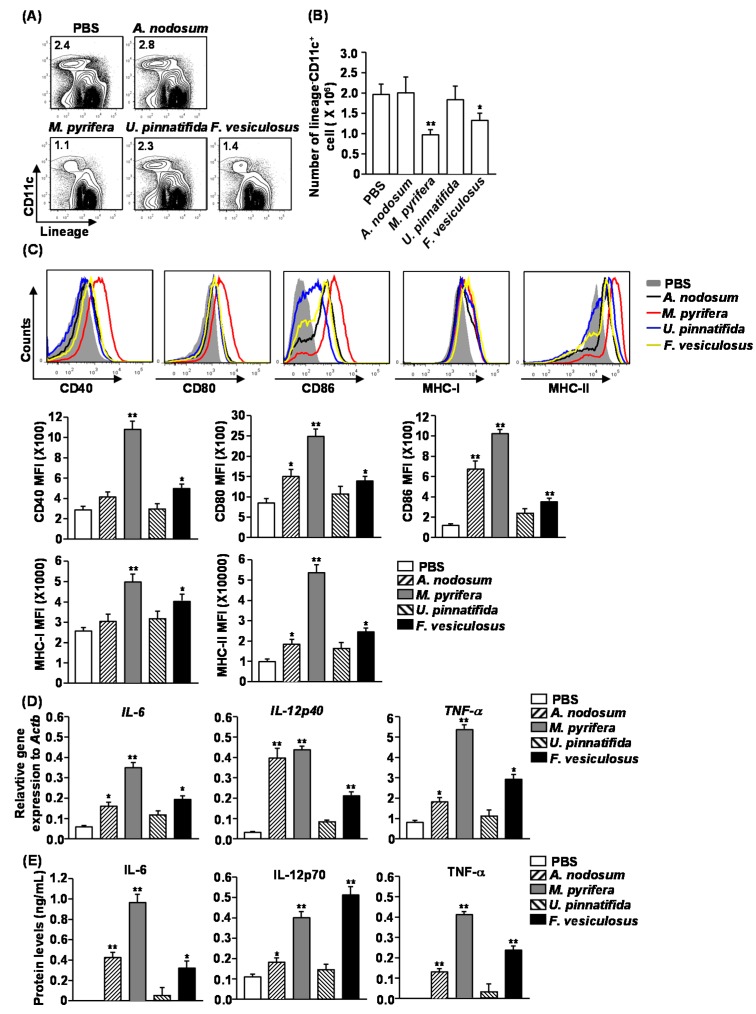
Administration of fucoidans induces spleen DC activation. C57BL/6 mice were injected *i.v.* with 50 mg/kg fucoidans for 24 h. (**A**) Percentage of DCs, defined as lineage^−^CD11c^+^, was analyzed by flow cytometry; (**B**) Absolute number of lineage^−^CD11c^+^ cells within live cells was shown; (**C**) Expression levels of CD40, CD80, CD86, MHC class I and MHC class II were measured by flow cytometry (upper panel). Mean fluorescence intensity (MFI) of these molecules is shown (lower panel). Data are representative of or the average of analyses of six independent samples (two mice per experiment, total of three independent experiments); (**D**) Expression levels of IL-6, IL-12p40 and TNF-α mRNA were measured from spleen 2 h after 50 mg/kg fucoidans injection; (**E**) IL-6, IL-12p70 and TNF-α concentrations in sera were shown from fucoidan-treated mice at 24 h after treatment. Data are representative of or the average of analyses of six independent samples (two mice per experiment, total of three independent experiments). Data shown are the mean ± SEM. * *p <* 0.05; ** *p <* 0.01 *versus* PBS group.

Our previous observation that fucoidan from *F. vesiculosus* promotes spleen DC activation prompted us to investigate the effect of different fucoidans on spleen DC activation *in vivo* [4]. We injected 50 mg/kg fucoidans *intravenously* (*i.v*.) to C57BL/6 mice and analyzed spleen DCs 24 h later. Treatment with fucoidans from *M. pyrifera* and *F. vesiculosus* led to a significant decrease in the proportion and numbers of spleen DCs, which were identified as lineage^−^CD11c^+^ cells, whereas fucoidans from *A. nodosum* and *U. pinnatifida* had no significant effect (Figure 3A,B). Moreover, all fucoidans induced substantial increases in the surface levels of CD80, CD86 and MHC class II in spleen DCs (Figure 3C). The most active stimulator was fucoidan from *M. pyrifera*, which induced up-regulation of CD40, CD80, CD86, MHC class I and MHC class II in spleen DCs (Figure 3C). The least active compounds were fucoidans from *A. nodosum* and *U. pinnatifida*, which did not induce up-regulation of CD40 and MHC class I (Figure 3C).

To determine whether fucoidans affect production of cytokines, we injected C57BL/6 mice with fucoidans and analyzed the production of pro-inflammatory cytokines after 2 or 24 h. *In vivo* administration of all fucoidans, except for that from *U. pinnatifida*, caused a marked increase in mRNA levels of IL-6, IL-12p40 and TNF-α in splenocytes 2 h post-injection compared to PBS-treated control mice (Figure 3D). Moreover, serum levels of IL-6, IL-12p70 and TNF-α were also dramatically increased in mice treated with fucoidans, except for those treated with fucoidan from *U. pinnatifida* (Figure 3E). Among the fucoidans, fucoidan from *M. pyrifera* had the strongest effect on promoting cytokine production. Therefore based on both the induction of co-stimulatory molecules and cytokine production, fucoidan from *M. pyrifera* was the most potent inducer of spleen DC activation and maturation *in vivo*.

### 2.5. Effect of Fucoidans on T Cell Activation and Differentiation in Vivo

**Figure 4:**
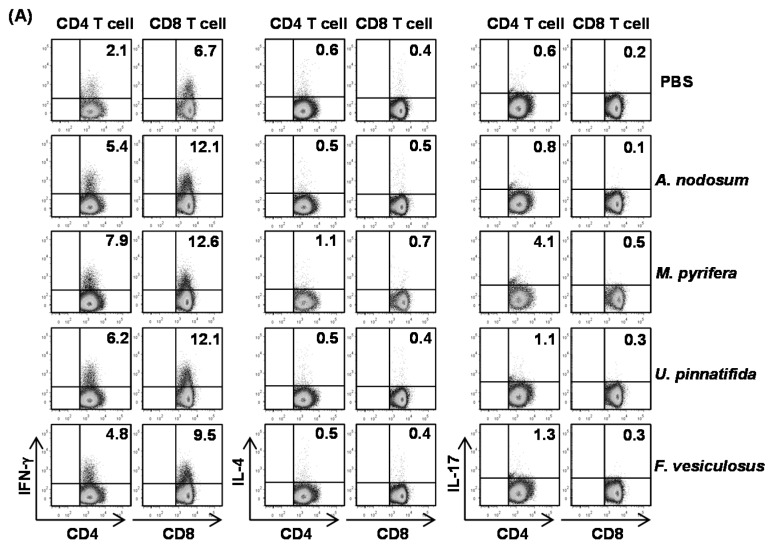

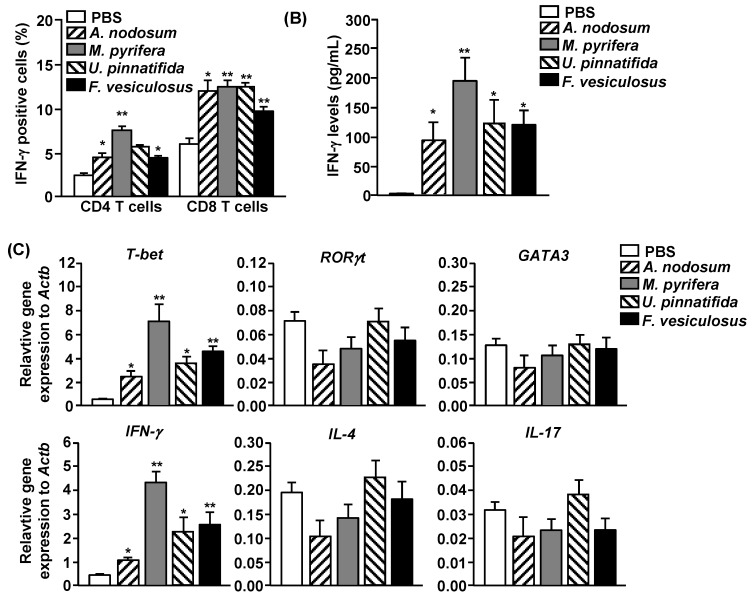
Fucoidans treatment increases IFN-γ-producing CD4 and CD8 T cells *in vivo*. C57BL/6 mice were injected *i.p.* with 50 mg/kg fucoidans and 3 days later, injected again with same amount of fucoidans. (**A**) Percentage of IFN-γ, IL-4 and IL-17 positive cells within CD4 and CD8 T cells, identified by gating on the CD4^+^CD8^−^ and CD4^−^CD8^+^ cells respectively, in the spleen was assessed by flow cytometric analysis (upper panel). Mean percentage of IFN-γ^+^ cells (lower panel); (**B**) IFN-γ concentrations in the serum. All data are representative of or the average of analyses of six independent samples (two mice per experiment, total of three independent experiments); (**C**) Gene expression in spleens was measured 24 h after fucoidan injection. Data are the average of analyses of six independent samples (two mice per experiment, total of three independent experiments). Data shown are the mean ± SEM. * *p <* 0.05; ** *p <* 0.01 *versus* PBS group.

To determine whether fucoidans can have an impact on T cell responses, we *i.p.* injected 20 mg/kg fucoidans to C57BL/6 and three days later, injected the same amount of fucoidans again. All fucoidan treatment led to marked increases in the proportions of splenic CD4 and CD8 T cells that produced IFN-γ and TNF-α, the signature cytokines of Th1 and Tc1 cells, as compared to PBS treatment (Figure 4A). In contrast, the percentages of IL-17- or IL-4-producing CD4 and CD8 T cells in the spleen were not significantly increased by fucoidans (Figure 4A). Serum levels of IFN-γ and TNF-α were also markedly increased by fucoidan (Figure 4B). Moreover, fucoidan-treated mice had significantly higher mRNA amounts of T-bet, the critical transcription factor for Th1 and Tc1 cells, and IFN-γ in the spleen than control mice (Figure 4C). In contrast, the mRNA levels of GATA3 and RORγt, transcription factor for Th2 and Th17, were not changed by fucoidan treatment (Figure 4C). Consistent with its effect on DC activation and maturation, fucoidan from *M. pyrifera* showed the strongest effect among the four fucoidans on T cell activation *in vivo*. Taken together, these results showed that fucoidan from *M. Pyrifera* is the most active immune modulator of NK cell, DC and T cell activation and function among the four fucoidans tested.

### 2.6. Effect of Fucoidans on Antigen Presentation by DCs and Antigen-Specific T Cell Proliferation

**Figure 5:**
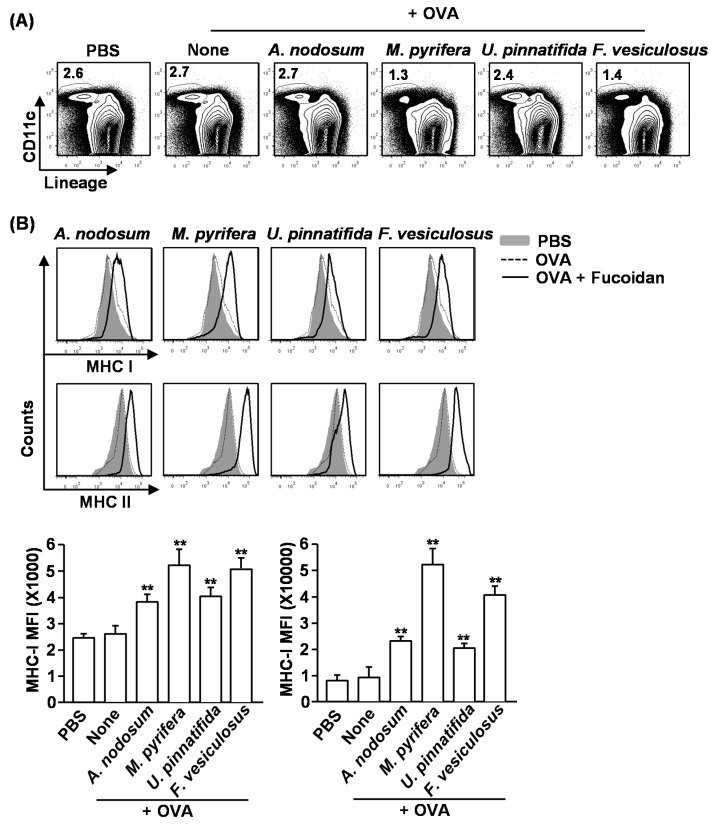

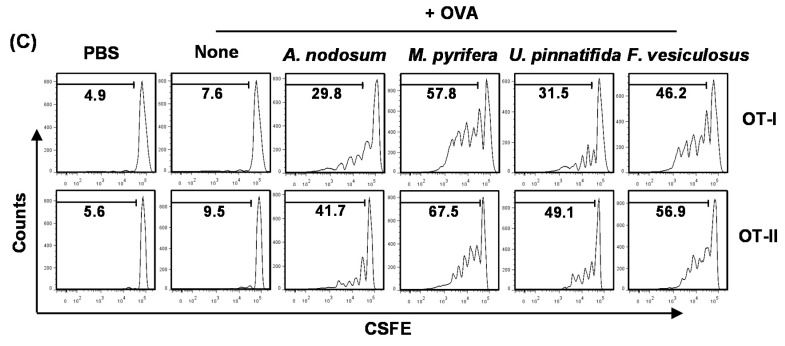
Fucoidans promote antigen-specific T cell proliferation *in vivo*. C57BL/6 mice were injected with PBS, ovalbumin (OVA) or OVA + fucoidan for 24 h. (**A**) Frequency of DCs, defined as lineage^−^CD11c^+^, were analyzed by flow cytometry; (**B**) Expression levels of MHC class I and II on the gated lineage^−^CD11c^+^ DCs in the spleen (upper panel). MFI of MHC class I and MHC class II is shown (lower panels); (**C**) Purified CD8 T cells from OT-I or CD4 T cells from OT-II mice were labeled with CFSE and transferred into CD45.1 congenic mice, and 24 h later, mice were injected with PBS, OVA or OVA + fucoidans. After 3 day treatment, splenocytes from these mice were stained for CD45.2 to identify the donor OT-I or OT-II cells and the proliferation of these cells was determined by CFSE dilution. All data are from analyses of six individual mice each group (two mice per experiment, total of three independent experiments). Data shown are the mean ± SEM. * *p <* 0.05; ** *p <* 0.01 *versus* OVA group.

Our finding that fucoidans induce spleen DC and T cell activation *in vivo* prompted us to further investigate the adjuvant effect of fucoidan in antigen-specific T cell response *in vivo*. We first examined whether fucoidans can promote antigen-presentation by DCs. Mice were injected with PBS, ovalbumin (OVA) or OVA + fucoidans for 24 h, and then measured for frequency of DCs and expression of MHC classes I and II on spleen lineage^−^CD11c^+^ DCs. Consistent with the results shown in Figure 3, the frequency of spleen CD11c^+^ DCs was dramatically decreased by injection of fucoidans from *M. pyrifera* or *F. vesiculosus* (Figure 5A). Moreover, all fucoidans promoted up-regulation of MHC class I and II expression on CD11c^+^ DCs (Figure 5B). As expected, fucoidan from *M. pyrifera* induced the greatest up-regulation of these surface proteins among the four fucoidans (Figure 5B). Next, we performed an adoptive transfer experiment to detect OVA-specific OT-I and OT-II T cell proliferation. CFSE-labeled OT-I CD8^+^ or OT-II CD4^+^ T cells from CD45.2 TCR-transgenic mice were transferred into CD45.1 congenic mice, and 24 h later, the mice received injection of PBS, OVA or OVA + fucoidans. After 3 days, the proliferation of CD45.2^+^ OT-I or OT-II cells was determined by CFSE dilution assay. OT-I and OT-II T cell proliferation was robustly increased in mice immunized with OVA + fucoidans compared to those in mice immunized with OVA alone (Figure 5C). Consistent with its effect on DC activation and maturation, fucoidan from *M. pyrifera* stimulated the strongest antigen-specific T cell proliferation. These data demonstrated that fucoidan functions as an adjuvant to enhance antigen presentation and antigen-specific CD4 and CD8 T cell activation. Moreover, fucoidan from *M. pyrifera* is the most effective adjuvant *in vivo* for antigen presentation among the four fucoidans tested.

### 2.7. Adjuvant Effect of Fucoidans on Antigen-Specific Immune Responses in Vivo

**Figure 6:**
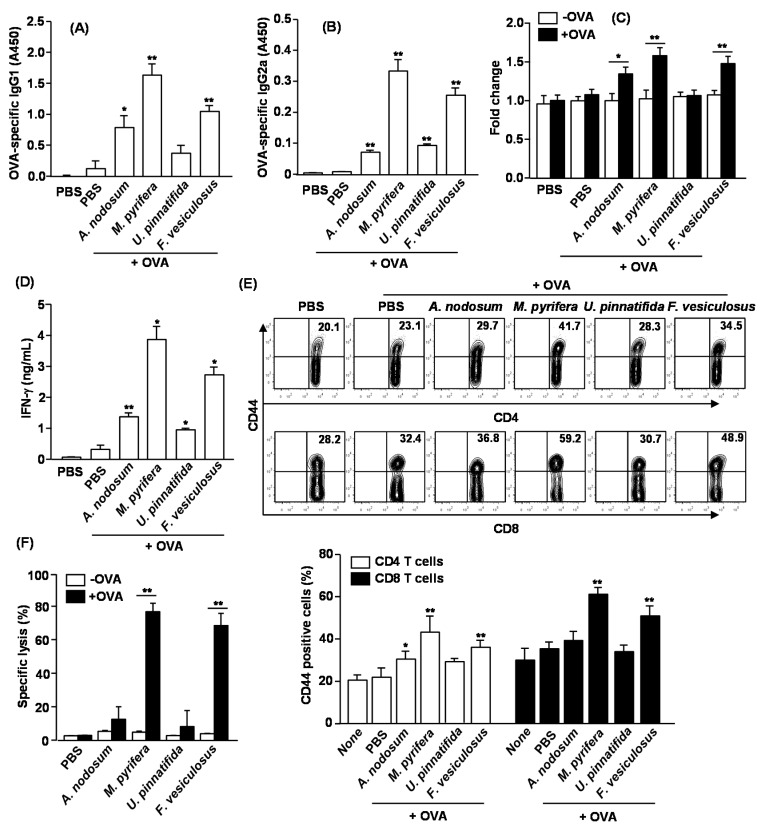
Immunization with OVA and fucoidans enhances OVA specific immunity. C57BL/6 mice were immunized *i.p.* with PBS, OVA or OVA + fucoidan on days 0, 14, 28. On Day 33, serum (**A**) OVA-specific IgG1 and (**B**) IgG2a concentrations were measured by ELISA. * *p <* 0.05; ** *p <* 0.01 *versus* OVA group; (**C**) Splenocytes were harvested from immunized mice on day 33, and re-stimulated with or without OVA (50 μg/mL) for 4 days. Cell proliferation from re-stimulated splenocytes was measured; (**D**) IFN-γ concentrations in the above splenocytes culture supernatants were shown; (**E**) CD44 expression on CD4 or CD8 T cells was analyzed by flow cytometry. The number in each plot indicates the percentage of CD44^+^ cells (upper panel). Mean percentage of CD44^+^ cells in CD4 or CD8 T cells was shown (lower panel). Data are representative of or the average of analyses of six independent samples (two mice per experiment, total of three independent experiments). * *p <* 0.05; ** *p <* 0.01 *versus* OVA group; (**F**) On Day 33, *in vivo* killing of adoptively transferred SIINFEK-coated and CFSE-labeled target cells by CTLs in the immunized mice was measured. Data are from analyses of six individual mice in each group (two mice per experiment, total of six independent experiments). * *p <* 0.05; ** *p <* 0.01.

To determine whether fucoidans exhibit adjuvant effect *in vivo* and which fucoidan is most effective, we immunized C57BL/6 mice with OVA and fucoidans, and examined the adjuvant effect including specific antibody production and T cell responses against OVA. C57BL/6 mice were injected *i.p.* with OVA alone or in combination with 50 mg/kg fucoidan on day 0, 14 and 28. On Day 33, sera were analyzed for OVA-specific IgG1 and IgG2a levels. Mice immunized with OVA + *M. pyrifera* fucoidan produced remarkably higher amounts of anti-OVA IgG1 and IgG2a than control mice immunized with OVA alone (Figure 6A,B). Fucoidan from *U. pinnatifida* showed less effect on anti-OVA IgG1 and IgG2a production. On day 33, splenocytes were also harvested, re-stimulated with OVA *in vitro* for four days, and analyzed for OVA-induced T cell proliferation, IFN-γ production and memory T cell generation. Splenocytes from mice immunized with OVA + *A. nodosum*, *M. pyrifera* and *F. vesiculosus* fucoidan showed significantly greater cell proliferation and IFN-γ production than those from control mice immunized with OVA alone (Figure 4C,D). These results indicate that fucoidans could function as an adjuvant by promoting Th1 type immune responses. We next examined whether fucoidans promote the generation of effector/memory T cells in OVA immunized mice based on the surface expression of CD44. Fucoidans from *A. nodosum*, *M. pyrifera* or *F. vesiculosus* led to marked increases in the proportions of CD44^+^ CD4 and CD8 T cells, whereas fucoidan from *U. pinnatifida* did not show such effect (Figure 6E). We also assayed for CTL activity in an *in vivo* cytotoxicity assay. On day 33 after the initial immunization, the immunized mice received SIINFEKL-pulsed and CFSE-labeled splenocytes from C57BL/6 donor mice. Higher than 70% of specific target cell lysis was observed in mice immunized with OVA + fucoidan from *M. pyrifera* or *F. vesiculosus*, indicative of T cell memory induction (Figure 6F). No significant target cell killing was observed in mice immunized with OVA + fucoidan from *A. nodosum* or *U. pinnatifida*. Collectively, these data suggest that fucoidans function as an adjuvant to enhance antigen specific T and B cell immune responses, and among the fucoidans tested in this study, fucoidan from *M. pyrifera* is the most effective adjuvant for induction antigen specific immune responses.

### 2.8. Discussion

Fucoidan, a sulfated polysaccharide purified from brown algae, has been reported to modulate immunity based on both *in vitro* and *in vivo* studies [3,4,17,18]. Although a variety of biological activities of fucoidan have been reported, immune modulation effect of different species of fucoidans has been not compared. In this study, we demonstrated that fucoidan from *M. pyrifera* has the most potent immune activating effect on human neutrophils, and on mouse NK cells, DCs and T cells compared to three other fucoidans.

Our previous study has shown that fucoidan from *U. pinnatifida* induces delay of apoptosis and production of pro-inflammatory cytokines in human neutrophils and may be potential therapeutic compounds for bacterial infectious diseases and neutropenia by controlling neutrophil homeostasis and function with fucoidan [13]. In this regard, we confirmed here that all fucoidans were able to delay spontaneous neutrophil apoptosis at high concentration. However, we demonstrated that only fucoidans derived from *U. pinnatifida* and *M. pyrifera* could inhibit neutrophil apoptosis at low concentration. The most plausible hypothesis to explain the specific inhibitory effect of fucoidans on neutrophil apoptosis could be their ability to react with L- and P-selectins on neutrophils [19]. It is well-established that L-selectin is efficiently shed from the surface of neutrophils and down-regulation of L-selectin is induced during programmed neutrophil apoptosis [20]. Fucoidan may block L-selectin shedding, which can contribute to the delay of neutrophil apoptosis. Whether fucoidans from *U. pinnatifida* and *M. pyrifera* can inhibit L-selectin shedding more efficiently than other fucoidans will be addressed in our future studies.

Activation of NK cells have been historically proposed as a complementary treatment for infectious diseases and cancer [21]. Interestingly, we found that different fucoidan shows different effect on NK cell proliferation and activation. Fucoidan from *U. pinnatifida* showed the strongest effect of NK cell expansion. However, fucoidan from *M. pyrifera* showed the strongest effect on the activation and cytotoxic activity of NK cells than that from *U. pinnatifida*. These differences may be caused by the ability of different fucoidans to induce different panels of cytokines *in vivo*. Since NK cell activation and proliferation are differentially regulated by different cytokines [22], fucoidan from *M. pyrifera* and *U. pinnatifida* may preferentially induce cytokines that promote NK cell activation or proliferation, respectively.

The present study also investigated the adjuvant effect of different fucoidans on immune responses in mice. An ideal vaccine adjuvant should boost cell-mediated immune responses, such as specific antigen presentation by DCs and antigen-specific T cell activation and proliferation, in order to effectively eliminate pathogens [23,24]. We showed that treatment with fucoidans from *M. pyrifera* and *F. vesiculosus* cause decreases in the number of spleen DCs. It has been shown that fully matured DCs can undergo apoptosis or anergy when they induced T cell activation [25,26]. Therefore, treatment of fucoidans from *M. pyrifera* and *F. vesiculosus* may induce full maturation of spleen DCs that in turn stimulate T cells, which can happen rapidly within 24 h of fucoidan treatment. Consistent with this, fucoidan from *M. pyrifera* and *F. vesiculosus* showed effective T cell stimulation effect compared with other fucoidans. Moreover, fucoidans from *M. pyrifera* and *F. vesiculosus* exhibit an effective adjuvant activity to facilitate soluble OVA antigen-induced Th1 and CTL responses, OVA-specific IgG1 and IgG2a production and memory T cell differentiation, consistent with previous report that crude extract of fucoidan from *F. vesiculosus* can functions as an adjuvant [4].

According to the previous reports, the bioactivities of fucoidan are depending on the sulfate content, and fucoidan with higher sulfate shows higher activity [16]. However, we found that fucoidan from *M. pyrifera* has the most effective function in activating NK cells, DCs and T cells and has the strongest adjuvant effect, while its sulfate content is not substantially difference from the other fucoidans. On the other hand, based on composition study, the uronic acid content in *M. pyrifera* fucoidan is higher than the other fucoidans. Interestingly, we have previously shown that ascophyllan purified from *A. nodosum* has higher uronic acid levels than fucoidan and can promotes stronger DC maturation and T cell activation than fucoidan [26,27]. Hence, the uronic acid levels in fucoidan may be a factor that determines the immune-activating capacity, at least the capacity of activating DCs and T cells. Further investigation of this possibility and elucidation of the molecular and structural basis determining the immune-modulatory activity of fucoidan will be crucial to the development and optimization of fucoidan-based therapeutics and adjuvants in future.

In this study, we investigated the immune modulatory effect of four fucoidans including their effect on the apoptosis of human neutrophil *in vitro* and activation of mouse NK, DC and T cells *in vivo*, as well as their adjuvant effect on antigen-specific immune responses. Our results provide evidence that the fucoidan derived from *M. pyrifera* is a powerful immune modulator, which can delay human neutrophil apoptosis, and enhance mouse NK cell activation, DC maturation, T cell immune responses, antigen specific antibody production and memory T cell generation. Fucoidan from *M. pyrifera* may be a potentially useful novel drug for infectious diseases and cancer, and an effective adjuvant for vaccine.

## 3. Experimental Section

### 3.1. Ethics Statement

This study was conducted according to the principles expressed in the Declaration of Helsinki. Elutriated neutrophils were obtained from healthy donors at the Shanghai Public Health Clinical Center. The Institutional Review Board at Shanghai Public Health Clinical Center approved this study (IRB number: 2012ZX09303013). Written informed consent was obtained from all volunteers.

### 3.2. Chemicals and Cytokines

Fucoidans from the powdered *A. nodosum* and *F. vesiculosus* was purified as described previously [17,27]. High purity of Fucoidans from *M. pyrifera* and *U. pinnatifida* were obtained from Sigma-Aldrich (St. Louis, MO, USA). Fucoidan solution was passed through an endotoxin-removal column (Detoxi-gel: Thermo Fisher Scientific, Waltham, MA, USA), and subsequently filtered through an endotoxin-removal filter (Zetapor Dispo: Wako, Osaka, Japan). The endotoxin levels in purified ascophyllan were evaluated using a Limulus amebocyte lysate (LAL) assay kit (Lonza, Basel, Switzerland). Fucoidans used in all experiments contained less than 0.1 endotoxin unit/mL.

### 3.3. Neutrophil Culture

Peripheral blood neutrophils were isolated from healthy young donors using a method involving dextran sedimentation and differential centrifugation through a 1.077 histopaque (Sigma) density gradient. The blood donors had not taken any anti-inflammatory drugs for at least three weeks prior to their sampling. Venous blood was collected on sodium citrate solution (3.8%). The cellular part of the blood was mixed with a solution of 3% dextran in 0.9% NaCl solution and kept for 45 min at 25 °C. The neutrophil-rich upper layer of the suspension was then collected and centrifuged (250 g, 10 min). Residual erythrocytes were removed by hypotonic lysis and the pellet so obtained was re-suspended in PBS. The suspension was centrifuged (250 g, 30 min) on Histopaque solution at 4 °C. Isolated neutrophils (2 × 10^5^/100 μL) were maintained in RPMI 1640 medium supplemented with 5% autologous serum, 1% glutamine, 100 U/mL penicillin, and 100 μg/mL streptomycin in 96-well flat-bottomed plates at 37 °C in a humidified atmosphere containing 5% CO_2_. Neutrophils were shown to be 95% pure morphologically by microscopy.

### 3.4. Morphological Assessment of Neutrophil Apoptosisb

Neutrophils were incubated in the presence or absence of fucoidan or various agents for 24 h, and then cells were spun down on a glass slide by a cytospin. Cells were fixed with methanol and stained with Giemsa staining solution. Percentages of apoptotic cells were determined by counting at least 300 cells per slide.

### 3.5. Annexin-V and Propidium Iodide (PI) Staining

Cultured cells were staining with Annexin V-FITC and PI in 100 μL of binding buffer for 15 min at room temperature (RT). After added 400 μL of binding buffer, the cells were analyzed by flow cytometry using a FACS aria II (Becton Dickinson, Franklin Lakes, NJ, USA).

### 3.6. Mice

C57BL/6 mice (6 weeks old) were purchased from the B&K Laboratory Animal Corp (Shanghai, China). OT-I and OT-II TCR transgenic mice and C57BL/6-Ly5.1 (CD45.1) congenic mice were obtained from Jackson Laboratory (Farmington, CT, USA), and kept under pathogen-free conditions. All experiments were carried out under the guidelines of the Institutional Animal Care and Use committee at the Shanghai Public Health Clinical Center (Shanghai, China). The protocol was approved by the committee on the Ethics of Animal Experiments of the Shanghai Public Health Clinical Center (Mouse Protocol Number: SYXK-2010-0098).

### 3.7. Antibodies

Isotype control antibodies (Abs) (IgG1, IgG2a or IgG2b), CD11c (HL3), CD4 (GK1.5), CD8α (YTS169.4), CD40 (3/23), CD80 (16-10A1), CD86 (GL-1), KLRG1 (MAFA) and NK 1.1 (PK136) were from BioLegend (San Diego, CA, USA); anti-MHC class I (AF6-88.5.3), anti-MHC class II (M5/114.15.2), anti-IFN-γ (XMG1.2), anti-IL-17 (TCC11-18H10.1) and anti-TNF-α (MP6-XT22) were from eBioscience (San Diego, CA, USA).

### 3.8. Flow Cytometry Analysis

Cells were washed with phosphate buffered saline (PBS) containing 0.5% BSA, pre-incubated for 15 min with unlabeled isotype control Abs, and then labeled with fluorescence-conjugated Abs by incubation on ice for 30 min followed by washing with PBS. Cells were analyzed on a FACS Aria II (Becton Dickinson) and FlowJo 8.6 software (Tree Star, Ashland, OR, USA). Cellular debris was excluded from the analysis by forward- and side-scatter gating. Dead cells were further excluded by 7 aminoactinomycin D (7AAD) (BioLegend) staining and gating on the 7AAD-negative population. As a control for nonspecific staining, isotype-matched irrelevant mAbs were used.

### 3.9. Natural Killer (NK) Cell Cytotoxicity Assay

YAC-1 cells were incubated with 15 μM calcein-AM for 30 min at 37 °C. After being washed with culture medium, cells were re-suspended in the medium and cultured for 2 h at 37 °C. After harvest and wash, YAC-1 cells were treated with 1.25 mM probenecid and mixed with CD3^−^NK1.1^+^ cells as effector cells to make 10:1 ratio of effectors to targets (E:T). After incubation for 4 h, cultured supernatant was harvested and mixed with 500 μL of 50 mM Tris-HCl, and measured fluorescence at 485 nm by spectrophotometer (Eppendorf, Hamburg, Germany). Percent specific lysis was calculated using the formula% specific calcein release = [(mean test release − mean spontaneous release)/(mean total release − mean spontaneous release)] × 100.

### 3.10. Spleen Dendritic Cell (DC) Analysis

Spleen DCs were analyzed according to an established method with modifications. Spleens were cut into small fragments and digested, with 2% fetal bovine serum (FCS) containing collagenase for 20 min at room temperature. Cells from the digest were centrifuged and the cell pellet was resuspended in 5 mL of 1077 histopaque (Sigma-Aldrich). More histopaque was then layered below the cell suspension, with EDTA-FCS-layered above it. After centrifugation at 1700 *g* for 10 min, the light density fraction (<1.077 g/cm^3^) was collected and incubated for 30 min with the following FITC-conjugated monoclonal antibodies (mAbs): anti-CD3 (17A2), anti-Thy1.1 (OX-7), anti-B220 (RA3-6B2), anti-Gr1 (RB68C5), anti-CD49b (DX5) and anti-TER-119 (TER-119). Cells were analyzed on a FACS Aria II (Becton Dickinson). The cDCs were identified as lineage^−^CD11c^+^ cells.

### 3.11. Ex Vivo T Cell Stimulation and Intracellular Cytokine Staining

Singles cells prepared from spleens were stimulated *in vitro* for 4 h with phorbol 12-myristate 13-acetate (50 ng/mL) and ionomycin (1 μM; both from Calbiochem, Billerica, MA, USA), with the addition of monensin solution (Biolegend) during the final 2 h. Cells were then stained for surface markers. For intracellular cytokine staining, cells were stained for surface molecules first, then fixed and permeabilized with Cytofix/Cytoperm buffer (eBioscience) and subsequently incubated with anti-cytokine antibodies in Perm/Wash buffer (eBioscience) for 30 min. Control staining with isotype control IgGs was performed in all experiments.

### 3.12. ELISA

IL-6, IL-8 and TNF-α protein levels in neutrophil cultured medium and IL-6, IL-12p70, IL-23 (p19/p40) and TNF-α concentrations in the sera were measured in triplicate using standard ELISA kits (Biolegend).

### 3.13. Real-Time qPCR

Total RNA was reverse-transcribed into cDNA using Oligo (dT) and M-MLV reverse transcriptase (Promega, Madison, WI, USA). The cDNA was subjected to real-time PCR amplification (Qiagen, Venlo, Limburg, Netherlands) for 40 cycles with annealing and extension temperature at 60 °C, on a LightCycler 480 Real-Time PCR System (Roche, Basel, Switzerland). Primer sequences are: mouse β-Actin forward, 5'-TGGATGACGATATCGCTGCG-3'; reverse, 5'-AGGGTCAGGATACCTCTCTT-3', IL-6 forward, 5'-AACGATGATGCACTTGCAGA-3'; reverse, 5'-GAGCATTGGAAATTGGGGTA-3', IL-12p40 forward, 5'-CACATCTGCTGCTCCACAAG-3'; reverse, 5'-CCGTCCGGAGTAATTTGGTG-3', TNF-α forward, 5'-CCTTTCACTCACTGGCCCAA-3'; reverse, 5'-AGTGCCTCTTCTGCCAGTTC-3', T-bet forward, 5'-CAACAACCCCTTTGCCAAAG-3'; reverse, 5'-TCCCCCAAGCATTGACAGT-3', GATA3 forward, 5'-CGGGTTCGGATGTAAGTCGAGG-3'; reverse, 5'-GATGTCCCTGCTCTCCTTGCTG-3', RORγt forward, 5'-CCGCTGAGAGGGCTTCAC-3'; reverse 5'-TGCAGGAGTAGGCCACATTACA-3', IFN-γ forward, 5'-GGATGCATTCATGAGTATTGC-3'; reverse, 5'-CTTTTCCGCTTCCTGAGG-3', IL-4 forward, 5'-ACAGGAGAAGGGACGCCAT-3'; reverse 5'-GAAGCCCTACAGACGAGCTCA-3', IL-17A forward, 5'-GCGCAAAAGTGAGCTCCAGA-3'; reverse 5'-ACAGAGGGATATCTATCAGGG-3'.

### 3.14. Mouse Immunization

C57BL/6 mice were immunized *i.p.* with PBS alone, 50 μg of OVA in PBS or OVA mixed with 50 mg/kg fucoidans in PBS on days 0, 14 and 28. On day 33, mice were sacrificed, sera were collected, and splenocytes were harvested for further analysis.

### 3.15. Ovalbumin (OVA)-Specific Antibody Analysis

Ninety-six well plates were coated with OVA (10 μg/mL) and blocked with 1% bovine serum albumin (BSA). Serum samples were diluted and added to each well, followed by incubation with biotin-conjugated anti-mouse IgG1 and IgG2a (Biolegend) and streptavidin-conjugated HRP. The reaction was developed by TMB substrate (Sigma), and A650 was measured using a plate reader.

### 3.16. OT-I and OT-II T Cell Proliferation

CD4 T cells from OT-II mice or CD8 T cells from OT-I mice were isolated from spleens using CD4 T cell or CD8 T cell isolation kit (Miltenyi Biotec), respectively. The cells were suspended in PBS/0.1% BSA containing 10 μM CFSE (Invitrogen) for 10 min. CFSE-labeled cells (1 × 10^6^) were *i.v.* transferred into CD45.1 congenic mice, and 24 h later, mice were injected with PBS alone, 50 μg of OVA in PBS or OVA plus fucoidan (10 mg/kg) in PBS. At 72 h after immunization, splenocytes were harvested and OT-I or OT-II T cell proliferation was determined by analyzing the CFSE fluorescence intensity through flow cytometry.

### 3.17. In Vivo Cytotoxicity Assay

Mice were injected *i.v.* with a mixture of splenocytes differentially labeled with CFSE (2, 20, or 200 nM) and loaded with 1, 10, or 100 nM SIINFEKL peptide, respectively, and spleen cells labeled with 10 mM CellTrackerTM Orange CMTMR (Life technologies, Carlsbad, CA, USA) and not loaded with peptide. A total of 10 × 10^6^ cells per mouse were injected, consisting of a mixture containing each target cell population. Splenocytes were collected 24 h after injection of target cells. Presence of viable target cells was determined using exclusion by 7-aminoactinomycin D. Percentage killing was calculated using the formula as described [28].

### 3.18. Statistical Analysis

Results are expressed as the mean ± standard error of the mean (SEM). Statistical significance was determined by Student’s *t*-test (two-tailed, two-sample equal variance). *p*-Values smaller than 0.05 were considered as statistically significant.

## 4. Conclusions

In conclusion, our results provide evidence that the fucoidan produced by *M. pyrifera* is a powerful immune modulator, which can enhance NK cell activation, DC maturation, CTL activity, Th1 immune responses, antigen specific antibody production and memory T cell generation. Fucoidan from *M. pyrifera* may be a potentially useful therapeutic agent for infectious diseases, cancer and effective adjuvant for vaccine.
